# Essential genes: a cross-species perspective

**DOI:** 10.1007/s00335-023-09984-1

**Published:** 2023-03-10

**Authors:** Pilar Cacheiro, Damian Smedley

**Affiliations:** William Harvey Research Institute, Queen Mary University of London, London, UK

## Abstract

Protein coding genes exhibit different degrees of intolerance to loss-of-function variation. The most intolerant genes, whose function is essential for cell or/and organism survival, inform on fundamental biological processes related to cell proliferation and organism development and provide a window on the molecular mechanisms of human disease. Here we present a brief overview of the resources and knowledge gathered around gene essentiality, from cancer cell lines to model organisms to human development. We outline the implications of using different sources of evidence and definitions to determine which genes are essential and highlight how information on the essentiality status of a gene can inform novel disease gene discovery and therapeutic target identification.

## Defining essentiality, the first challenge

Knowing how essential a gene is can be key to understanding its function and potential involvement in disease. A gene’s function may be required for cell proliferation and fitness (cellular gene essentiality) or for growth and development of an organism (organismal gene essentiality), where its disruption may lead to lethality ranging from the embryonic stage to any period before reproductive age (Rancati et al. 2018).

Most viability studies in mammalian model organisms evaluate embryonic and/or postnatal viability, and these, together with cell proliferation assays, are some of our main sources of essential gene data. When we try to translate this knowledge to humans, mainly for the study of human genetic disorders, these distinctions and the resulting variability in the number of genes labelled as essential are especially relevant. In particular, lethal genes or disorders may include those associated with pre-reproductive lethality or where reproduction is impaired, the latter due to biological reasons or physical and intellectual phenotypes that impede reproductive success (Gao et al. 2015; Amorim, et al. 2017).

Hence, finding which genes are essential is subject to the criteria we use to define essentiality and this constitutes one of several hurdles. Adding to this challenge, regardless of the interpretation of essentiality and even considering the same organism or level of organisation, gene essentiality is not an unconditional feature. A gene may only be found to be essential in certain cell lines, or in some individuals within the same species (i.e. mouse genetic backgrounds) implying that the interpretation of essentiality as a binary attribute is a simplification of a more complex and highly context-dependent feature (Rancati et al. 2018; Sharma et al. 2020).

## What is the actual number of essential genes?

An increasing number of large scale CRISPR and RNAi screens are being conducted in human cancer cell lines: The Cancer Dependency Map (DepMap) and Project Score are two of the main projects aimed at investigating the effects of gene knockout on the fitness of cell lines, providing scores that indicate cell growth inhibition or death (Meyers et al. 2017; Dwane et al. 2021). A given gene may show variable essentiality scores across cell lines, and several attempts have been made to identify a core set of essential genes. However, the criteria and selected score thresholds can differ between studies. CEN-tools selected a cluster of genes with a high probability of being essential across all the investigated cell lines from the projects mentioned above, with 942 and 650 genes, respectively, assigned to this core essential gene cluster, 519 of which were shared between the two projects (Sharma et al. 2020). Previous screens on a lower number of cell lines independently reported 1878, 1734 and 1580 core fitness/essential genes (Wang et al. 2015; Blomen et al. 2015; Hart et al. 2015), although the exact number may again depend on the selected cut-off. According to these results, around 8–10% of protein coding genes would be considered cellular essential. A more recent resource reveals the phenotypic consequences of disrupting core cellular processes and provides a new landscape of human cellular essential genes, including multidimensional image-based phenotypes for 5072 genes. These genes have been identified to contribute to optimal cellular fitness, but the evidence comes from multiple genetic screens including the ones mentioned above, and not all these genes are necessarily essential in all the cell lines. These would comprise ~ 25% of the coding genome (Funk, et al. 2022).

We are halfway to completing a map of essentiality or viability in the mouse. Even accounting for the limitations of orthology mapping, we can see important differences in the number of lethal lines and the percentage of the coding genome considered essential compared to cellular screening approaches. In the mouse, this percentage increases to 35% of investigated genes based on an International Mouse Phenotyping Consortium (IMPC) standardised viability screen (Peterson and Murray 2021), a percentage that remains stable across data releases (Groza et al. 2022), with 2583 out of 7335 genes assessed as lethal (1804, 24.6%) or subviable (779, 10.6%) according to release 18.0. When using Mouse Genome Informatics (MGI) phenotypic annotations from lines with heterogeneous backgrounds (Bult et al. 2019), that percentage rises to 39%, with 4956 out of 12,753 genes having an associated lethal phenotype according to a selected set of phenotype terms ranging from embryonic lethality before implantation (embryonic day (E) 0 to less than E4.5) to postnatal/weaning stage (3–4 weeks of age) (Dickinson, et al. 2016). Discrepancies in lethality between mouse knockouts from the IMPC and other models collated by MGI can be found in ~ 10% of the lines (Cacheiro et al. 2020). The impact of genetic background on the particular phenotype of essentiality has been investigated in different model organisms (Hou et al. 2019; Bello et al. 2020). Additionally, within one species gene essentiality may be ‘bypassable’ by monogenic suppressors (Li et al. 2019; van Leeuwen, et al. 2020). Different categorisations of levels of essentiality have also been described in yeast: conditional essential, essential, redundant essential and absolute essential (Zhang and Ren 2015). Regardless of whether we consider essentiality at the cellular level (gene essential for cell proliferation/fitness/survival) or at the organism level (gene essential for development), gene essentiality cannot be considered a simple binary or static attribute^1^.

**Fig. 1:**
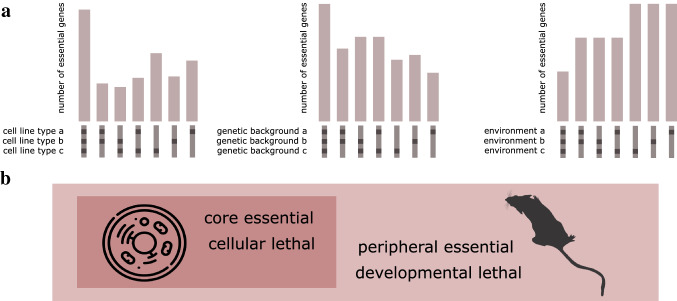
Essential gene classifications **a** Essentiality may be cell type/tissue specific, i.e. some genes are essential in all cell lines, some essential genes are only shared by certain lines or tissues and some others may be only essential in one particular tissue. Similarly, for different genetic backgrounds or environments; **b** Combining information from different sources of evidence (knockout screens in human cell lines and mouse) has led to a distinction between two main sets of essential genes. The boundaries between these categories may not be clearly defined, and the set of ‘core’ or ‘cellular’ essential genes can consenquently differ depending on the criteria and cut-offs selected

A subdivision into two main sets of essential genes has been made in different studies. Hart et al. defined these sets as ‘core essential’ (essential in all cell lines and contexts) and ‘peripheral essential’ (tissue/context-specific essential) (Hart et al. 2014). Cacheiro et al. defined genes as either ‘cellular lethal’ or ‘developmental lethal’, the first set comprising a set of genes essential for cell proliferation in most cell lines, and the second one those genes where the mouse knockout results in preweaning lethality, and that are not included in the previous set (Cacheiro et al. 2020). Each category contains 35% and 65% of all lethal genes, respectively. Unsurprisingly, when the developmental stage at which the knockout mice die is considered, early gestation lethal genes, where the mouse embryo dies before embryonic day E9.5, highly correlate with cellular lethal genes (Cacheiro et al. 2022). This indicates that there is a set of genes that are essential in most cell lines and/or common to all tissues and contexts and constitutively expressed that are involved in regulating basic cellular functions and another set of essential genes that may be tissue, context or developmental stage specific (Fig. 1).

There are several web portals available that capture information on gene essentiality from different resources and organisms. The Database of Essential Genes (DEG) comprises a catalogue of essential genes identified in bacteria and eukaryotes (Luo et al. 2021). The set of human essential genes comes from three different sources: human orthologues of mouse essential genes; genes with high probability of intolerance to loss-of-function (LoF) variation from the Genome Aggregation Database (gnomAD) (Karczewski et al. 2020); and genes essential according to large scale screenings in human cell types. Similarly, regarding essential genes in humans, only CRISPR-Cas9 and RNAi experimental data in cancer cell lines is included in the Online GEne Essentiality Database (OGEE), another online resource containing essentiality data from 91 species, where conditional (context-dependent) essential genes, i.e. those whose essentiality status differs across datasets, are tagged (Gurumayum et al. 2021).

## Genes essential for human development

Notably, and as reflected by the sets of genes collated in the DEG database, our evidence on human essential genes comes from cellular assays, human orthologues of mouse knockouts and different intolerance scores derived from human population sequencing data. Two considerations need to be factored in when using these intolerance to LoF variation scores in this context: (1) they are primarily aimed at identifying pathogenic variants and disease-associated genes and not characterisation of essential genes, (2) they capture dominant effects, as opposed to mouse models where intolerance to homozygous LoF variation is being evaluated (Bartha et al. 2018).

**Fig. 2:**
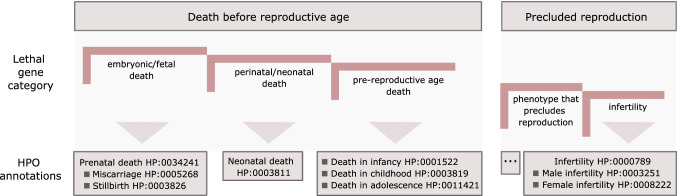
Genes essential for human organism development Defining a set of essential (lethal) genes in humans may depend on the purpose of the study. A subclassification of lethal genes is suggested based on the associated age of death according to Human Phenotype Ontology (HPO) terms and definitions

None of these sources of data fully captures those genes that are essential for human organism development, i.e. those genes with evidence of LoF leading to prenatal or neonatal death. This could also include those genes associated with pre- reproductive death, or causing severe impact on fitness that precludes reproductive success or infertility. As mentioned above, the definition and consequent set of genes essential for human development may vary depending on the purpose of the study (Amorim, et al. 2017). A solution could be to classify the set of known lethal genes in humans according to the developmental stage at which lethality occurs, as defined by the Human Phenotype Ontology (HPO) age of death phenotype terms and descriptions (Kohler et al. 2021) (Fig. 2).

Prenatal sequencing is being more widely implemented and allows the detection of severe problems, e.g. foetal structural anomalies, prior to birth, and in the extreme case of miscarriage or stillbirth, to perform molecular autopsies to clarify the genetic cause of the prenatal death (Robbins et al. 2019; Stanley et al. 2020; Colley et al. 2019; Yates et al. 2017; Lord et al. 2019). Similarly, whole genome or exome sequencing is being introduced to investigate the genetic causes of recurrent pregnancy loss (Najafi et al. 2021). First trimester abortuses with no chromosomal abnormalities—that constitute up to 50% of early pregnancy losses—could have a Mendelian origin or more complex pattern of inheritance. The risk of aneuploidy is lower for losses occurring later in the pregnancy (Hyde and Schust 2015). Additionally we could consider the aforementioned disorders, where death may occur later in life but before reproductive age, or with associated phenotypes that impede reproduction (Amorim, et al. 2017). Resources for Mendelian disorders documenting clinical reports that allow users to perform queries using a phenotypic approach include the Online Mendelian Inheritance in Man (OMIM) (Amberger, et al. 2019) and the HPO knowledge bases. However, the evidence for conditions with prenatal lethal phenotypes in humans is either very limited or not easy to retrieve from these repositories. One informatic toolkit extracted information from a number of various sources to produce a curated list of genes candidates to be linked to unexplained infertility and prenatal/infantile mortality (Dawes et al. 2019). Genes associated with infertility have been recently captured in a specific resource (Wu et al. 2021). Reduced fertility may indeed be associated with (recurrent) early pregnancy loss.

## Essential genes and human disease

The essentiality status of a gene provides information on the biological process, stages of development, cell types and/or tissues where the gene function is required. Additionally, they constitute a key resource to investigate the mechanisms of disease. The link between essential genes and human disorders is beyond doubt, with an overall enrichment of Mendelian disease genes amongst the set of lethal mouse orthologue genes (Dickinson, et al. 2016; Dickerson et al. 2011).

Those genes where homozygous LoF is known to cause embryonic or postnatal lethality in the mouse constitute potential candidates to be associated with early lethal phenotypes in humans (Dawes et al. 2019). Hypomorphic or non-LoF variants in those genes could lead to partial loss or altered function with different phenotypic manifestations, in addition to heterozygous LoF variation through dominant-negative or haploinsufficient effects. These may result in later onset and/or non-lethal phenotypes that explain the strong association between mouse lethal and human disease genes. There are multiple examples of homozygous mouse knockout lethal genes where heterozygous de novo variants cause neurodevelopmental disorders (Chao et al. 2017; Rodger et al. 2020; Cousin, et al. 2021). Prenatal lethality may be considered the most severe phenotypic manifestation of a Mendelian disorder observed among a much wider spectrum of clinical features (Alkuraya 2015; Shamseldin et al. 2015). Amongst those genes essential for human development with abnormal phenotypes observed during prenatal development stages, we find two different scenarios that potentially explain the underrepresentation of these genes in current gene-disease databases: (a) the associated phenotypes are restricted to foetal life, hence they may have eluded etiological and clinical characterisation since the outcome is invariably prenatal or perinatal death; (b) the prenatal manifestations constitute a more severe presentation of a phenotype previously only observed during postnatal stages, hindering the molecular diagnosis at this developmental phase (Meier et al. 2019). We expect to find a combination of genes where pre/perinatal lethality is the only observable phenotype and genes where early lethality is part of a wider, postnatal phenotypic spectrum.

This link between essential and disease-associated genes has been exploited to investigate particular types of disorders and for prioritising genes and potentially pathogenic variants in human sequencing studies. Ji et al. provided evidence that deleterious variants in essential genes contribute to autism spectrum disorder risk (Ji et al. 2016). Two other studies found distinct disease categories to be overrepresented in different subsets of mouse lethal genes, information that was leveraged to develop novel gene discovery strategies for neurodevelopmental disorders and inborn errors of the metabolism (Cacheiro et al. 2020; Cacheiro et al. 2022).

Machine learning approaches integrating gene expression across development in relevant tissues among other features are providing prioritised sets of genes to be associated with developmental disorders (Dhindsa et al. 2022). The Fetal Sequencing Consortium and specific sequencing programmes aimed at providing a molecular diagnosis for this type of disorders (e.g. Deciphering Developmental Disorders) are identifying potentially pathogenic variants in genes not yet associated with disease (Giordano and Wapner 2022; Deciphering Developmental Disorders Study 2015). These, together with the known set of Mendelian disease genes, those genes essential in cell lines, and the human orthologues of mouse and other model organism lethal genes constitute our most reliable sources of information to identify novel candidate genes and catalogue all the genes crucial for human development.

## Essential genes and drug targets

The screens performed across hundreds of different human cancer cell lines allow the generation of cancer dependency maps, since individual cancers may depend on distinct essential genes for their proliferation and/or survival. These conditionally essential genes with variable essentiality across tissues allow the detection of vulnerabilities and provide valuable information to identify promising therapeutic targets, potentially informing on efficacy, selectivity and toxicity (Sharma et al. 2020; Shimada et al. 2021). 

Information on embryonic and postnatal lethality, together with other abnormal phenotypes observed in the mouse knockout, is currently being used in different platforms aimed at identifying and prioritising potential drug targets (Ochoa et al. 2022; Nguyen et al. 2017).

The interpretation of constraint metrics derived from human sequencing studies and the identification and phenotypic characterisation of carriers of LoF variants can also assist drug target identification (Minikel et al. 2020). Not only the genes where the LoF has a strong phenotypic impact are of interest, but also those at the other end of the spectrum, where a naturally occurring LoF variant has no apparent or subtle impact on the phenotype or may prove even beneficial to health. The study of these ‘human knockouts’ is key for drug development as they may inform on drug efficacy and safety (Narasimhan et al. 2016).

## Conclusion

Existing comprehensive resources are collating information on cellular essentiality across a large number of human cancer cell lines. Similarly, information from large human sequencing programmes is used to infer metrics of gene intolerance to LoF variation. Information on gene essentiality in the mouse orthologue is available for up to 2/3 of human protein coding genes. These different lines of evidence provide invaluable information for the diagnosis and understanding of rare genetic disorders and the identification of therapeutic targets. However, the catalogue of genes that are essential for human development is not yet complete. These datasets, together with the known and candidate set of Mendelian genes and their associated phenotypes, can be integrated to create a catalogue of genes essential for human development, ranging from those leading to embryonic lethal phenotypes to death before pre-reproductive age. This catalogue will be indispensable for our future efforts in diagnosing and treating patients with rare conditions and understanding the causes of miscarriage and reduced fertility.

## Data Availability

All the data presented in the manuscript is available in the references provided. Detailed IMPC and MGI mouse viability data is publicly available through their respective web portals: http://ftp.ebi.ac.uk/pub/databases/impc/all-data-releases/release-18.0/results/viability.csv.gz and http://www.informatics.jax.org/downloads/reports/MGI_GenePheno.rpt
